# Biosimilars in practice: Discriminant factors influencing prescription decisions among physicians in Thailand

**DOI:** 10.1371/journal.pone.0327591

**Published:** 2025-07-03

**Authors:** Chaoncin Sooksriwong, Ekapong Kachai, Wanruchada Katchamart, Ponlapat Rojnuckarin, Ekaphop Sirachainan, Tuangrat Phodha

**Affiliations:** 1 Drug Information and Consumer Protection Center, Center of Excellence in Pharmacy Practice and Management Research, Faculty of Pharmacy, Thammasat University, Rangsit Campus, Pathum Thani, Thailand; 2 Division of Rheumatology, Department of Medicine, Faculty of Medicine Siriraj Hospital, Mahidol University, Bangkok, Thailand; 3 Division of Hematology, Department of Medicine, Faculty of Medicine, Chulalongkorn University, Bangkok, Thailand; 4 Oncology Unit, Department of Medicine, Faculty of Medicine Ramathibodi Hospital, Mahidol University, Bangkok, Thailand; Shanghai Jiao Tong University Medical School Affiliated Ruijin Hospital, CHINA

## Abstract

This study aimed to assess the discriminant factors and determine the cutoff value which can predict or classify the group of prescribed and non-prescribed biosimilar among Thai physicians. The online surveys were distributed to physicians via major three medical associations in Thailand. Five psychological variables were obtained from the surveys (familiarity, attitude toward biosimilar medication, attitude toward biosimilar practice scenarios, attitude toward naming biosimilars in prescriptions, and attitude toward pricing of biosimilar). The point-based system was used to score all variables and transformed to percentage. The assumptions were tested before using discriminant function analysis (DFA). Total 82 respondents were analyzed. Data of all variables met the assumptions of DFA. Familiarity was the most influential factor to differentiate the group of physicians, followed by attitude toward biosimilar medication. The cutoff value for group differences was −0.600. The accuracy rate of discriminant function equations was 82.9% overall for the stepwise method. The study concluded that the psychological factors such as familiarity with biosimilars and attitude toward biosimilars would play a significant role to classify between prescribed and non-prescribed biosimilar groups.

## Introduction

Globally, the biosimilars market is expanding rapidly. According to market research, the market value of biosimilars is projected to reach 76.2 billion USD by 2030 [1], up from an estimated 27.2 billion USD in 2023. Biosimilars are more appealing to both healthcare payers and patients due to their advantage to reduce healthcare expenditures, particularly in the treatment of complex chronic diseases requiring expensive biologic medicines, such as diabetes, autoimmune diseases, and cancers.

Biosimilar can be introduced after the reference biologic drug’s (also known as the originator biologic drug) patent expired. It is approved as being comparable to the originator biologic drug in terms of quality, safety, and efficacy, but at a lower price [2,3]. To maintain biosimilar adoption and penetration nationally, the appropriate policies must be enacted. Government policies play a crucial role in determining the trajectory of biosimilars market in that country. The policies governing the adoption of biosimilars should be shaped by supply-side and demand-side policies to support the short- and long-term adoption of biosimilars [4,5]. Despite this, studies have found that the familiarity and attitudes of healthcare professionals regarding biosimilars vary by region [6,7]. Some studies revealed a low level of familiarity, while others revealed a good level of knowledge and a positive attitude among healthcare professionals; however, they were unwilling to prescribe or switch. Thus, understanding the factors affecting biosimilar adoption is crucial.

In Thailand, the Thai FDA issued its first biosimilar registration guidance in 2013 and a reversion in 2018 [3]. These guidelines have significantly influenced biosimilar adoption by ensuring safety, efficacy, and quality. Moreover, they have reduced barriers to market entry and increased competition in the biologics market. According to the Thai FDA database, the import value of biosimilars increased from 1.4 billion to 3.3 billion Thai Baht between 2019–2023 [8]. This partially implies the effectiveness of the implementation of policies related to the availability and utilization of biosimilars, such as the reversion of biosimilar registration guidance by the Thai FDA in 2018 [9], the “price-performance criteria” for tender bidding of pharmaceutical products based on the Government Procurement and Supplies Management Act B.E. 2541 (2017) [10], and the announcement in 2022 from The Comptroller General’s Department (CGD), Ministry of Finance, which requested for cooperation from healthcare facilities to procure quality biosimilars and essential medicines at prices within the established reimbursement limits.” [11].

The policy enforcement of the Thai government may lead to the expansion of the biosimilars market, but it primarily influences the use of biosimilars in the public sector, not private sector. To encourage biosimilars uptake among the physicians in Thailand in overall, the factors such as familiarity, attitude toward prescribing biosimilars should be evaluated. Despite growing research on factors influencing biosimilar prescribing, most prior studies have usually employed descriptive statistics or logistic regression models to identify associations, rather than to effectively classify prescribing behaviors [12–15]. These approaches may lack the discriminative power to distinguish between physicians who adopt biosimilars and those who do not. To address this gap, the present study applied discriminant function analysis (DFA), a parametric classification technique, to categorize cases into distinct groups based on predictor variables. DFA not only identifies significant differentiating factors but also constructs a predictive model capable of accurately classifying physician behavior. Thus, the purpose of this study is to identify the key factors which distinguish between prescribed and non-prescribed biosimilar group among Thai physicians and to develop the discriminant function that could classify the physicians into either groups by using a decision point. Understanding these factors can assist payers, policymakers, and other stakeholders in developing policies that can effectively support the adoption of biosimilars in the private and public sectors of Thailand’s healthcare system.

## Materials and methods

### Study design and setting

This study was a cross-sectional, online survey of physicians in Thailand. The physicians who were members of rheumatology, oncology, or hematology association were eligible to participate, as these specialties frequently prescribe biologics and biosimilars and manage large patient groups, making them highly relevant to the study’s objectives. This ensured that the responses reflect real-world practice and insights from those most impacted. The physicians who could not read and understand the Thai language, or who did not complete the survey, were excluded from this study.

### Target population and sampling

This study utilized a total population of 1,276 physicians from the major three medical associations: the Thai Rheumatism Association, the Thai Society of Clinical Oncology, and the Thai Society of Hematology [16]. In this research, the sample size was calculated using Cochran’s formula. According to this formula, z = 1.96 at a confidence percentage of 95%, a margin of error of 5%, and expected sample proportion of 50% are taken into account. As a result, the sample size of this study was 296 physicians. This study used the convenience sampling approach to distribute the survey to sample group. Other medical associations were excluded because the biosimilars prescribed by other specialties were expected to be minimal.

**Cochran’s formula**


$$\text{Sample}size=\frac{\frac{z^{2}\times p(1-p)}{e^{2}}}{1+\left(\frac{z^{2}\times p(1-p)}{e^{2}N}\right)}$$

where N = Total population, e = Margin of error (percentage in decimal form), z = z-score, p = expected sample proportion, q = 1-p.

### Ethical consideration

The study was approved by The Human Research Ethics Committee of Thammasat University (Science) (COA No. 085/2565). All voluntary respondents gave informed consent by checking a compulsory informed consent field before accessing the questionnaires.

### Survey questions design and evaluation

The questionnaire was designed based on literature review. Its content validity was evaluated by three experts. Each item was evaluated using the Item-Objective Congruence (IOC) method and items scoring below the acceptable threshold (below 0.67) were revised based on experts’ feedback. Due to previous literatures indicated a low response rate and limited accessibility of physicians, a separate pilot study was not conducted [17–19]. Instead, the survey was refined through expert validation and IOC analysis to ensure clarity and content validity, while preserving the full sample for the main study. Importantly, physicians from relevant fields were involved in the design process, enhancing contextual relevance and practical applicability. There were 3 parts in the survey: 1) demographic background; 2) questions related to familiarity with biosimilars; and 3) attitude toward biosimilars. The 6 demographic variables were gender, age, years of experience working as a physician, specialty, self-assessment of biosimilar familiarity, and the experience of prescribing biosimilars.

A point-based system, depending on the number of correct answers, was used to calculate the score for familiarity variable (*X1*). This variable reflected respondents’ knowledge of biosimilar, covering the key topics such as definition, molecular characteristics, regulatory approval process, immunogenicity, extrapolation of indications, switching, and automatic substitution concepts. A correct answer scored 1 point, whereas 0 point was given for an incorrect answer. The ‘Don’t know’ answer was included to reduce the guessing effect and was scored as incorrect.

For the eleven attitude questions, the responses were grouped into four variables as follows:

Biosimilar medication *(X2):* This variable reflected attitudes towards the efficacy and safety of biosimilars, their indications, approval process of Thai FDA, importance, and benefits.

Practice scenarios (*X3*): This variable measured overall confidence in prescribing biosimilars across five different scenarios: for a naive patient; for a patient with a stable condition who has previously used the reference biologic; for a patient intolerant to the reference biologic; for a patient with a non-stable condition who has used the reference biologic; and for a patient with a history of allergy to the reference biologic.

Naming biosimilars in prescriptions (*X4*): This variable assessed attitudes towards using either the trade name or the molecule name of biosimilars in prescriptions.

Pricing of biosimilar (*X5*): This variable represented attitudes towards the pricing of biosimilars in comparison with their reference drugs.

Regarding attitude questions, a 3-point Likert scale with ‘Agree’, ‘Disagree’, and ‘Don’t know’ options was employed. The scoring for the level of agreement was ‘1’ for ‘Agree’ and ‘0’ for ‘Disagree’. However, the score for negative questions was converted. To minimize the influence of guessing, the ‘Don’t know’ response scored as ‘0’. The raw scores from familiarity and attitude section were transformed and presented as percentages for the analysis (a full score = 100%). The original questionnaires were developed in Thai and are available upon request.

### Data collection

The online survey was performed using Google Forms. The survey’s link and QR code were distributed via email to Thai physicians from the three Thai medical associations. The survey was initially distributed between 1–30 November 2022, followed by a reminder email sent between 1–15 December 2022 to maximize response rates.

### Statistical analysis

The SPSS Statistics for Windows, version 28.0 (IBM Corp., Armonk, NY, United States) was employed for data analysis. The categorical variables were presented as frequency and proportion (%). The relationship between categorical variables was examined using the Chi-square or Fisher’s exact tests. A 2-sample t-test was performed to assess the differences between two groups for each continuous variable. The statistical significance level was set at *p*-value < 0.05.

Moreover, a discriminant function analysis (DFA) was used to identify which variables best classified the differences between prescribed and non-prescribed biosimilar groups. The discriminant function and the decision point were developed [20–22]. The assumptions of the discriminant analysis were examined prior to conducting DFA by using the stepwise method [23]. Press’s Q statistic (chi-square with one degree of freedom) was calculated to evaluate whether the function performs statistically better than a chance model [24].

## Results

### Demographics of respondents

Total of 82 respondents participated in this study. The response rate was 27.7% of the expected sample size. Table 1 provides the demographics of respondents across the different specialties.

**Table 1:** Demographic characteristics of respondents (n = 82).

Characteristics	Specialty				Total	*p*-value
	Rheumatology n = 26 (31.7%)	Oncology n = 18 (22.0%)	Hematology n = 28 (34.1%)	Others n = 10 (12.2%)		
**Gender**						0.002^a*^
** **Male	4 (15.4)	12 (14.6)	16 (57.1)	6 (60)	38 (46.3)	
** **Female	22 (84.6)	6 (33.3)	12 (42.9)	4 (40.0)	44 (53.7)	
**Age (years)**						0.580^b^
25 - 40	12 (46.2)	9 (50.0)	16 (57.1)	2 (20.0)	39 (47.6)	
41 - 50	8 (30.8)	4 (22.2)	6 (21.4%)	4 (40.0)	22 (26.8)	
51 - 65	6 (23.1)	5 (27.8)	6 (21.4)	4 (40.0)	21 (25.6)	
**Experience as a physician (years)**						0.637^b^
≤ 10	6 (23.1)	7 (38.9)	12 (42.9)	2 (20.0)	27 (32.9)	
11-15	6 (23.1)	3 (16.7)	5 (17.9)	1 (10.0)	15 (18.3)	
≥ 16	14 (53.8)	8 (44.4)	11 (39.3)	7 (70.0)	40 (48.8)	
**Self-assessment familiarity**						0.000^b*^
Not familiar	4 (15.4)	1 (5.6)	4 (14.3)	10 (100.0)	19 (23.2)	
Familiar	19 (73.1)	11 (61.1)	14 (50.0)	0 (0.0)	44 (53.7)	
Very familiar	3 (11.5)	6 (33.3)	10 (35.7)	0 (0.0)	19 (23.2)	
**Experience of prescribing biosimilars**						0.000^b*^
No	3 (11.5)	1 (5.6)	3 (10.7)	10 (100.0)	17 (20.7)	
Yes	23 (88.5)	17 (94.4)	25 (89.3)	0 (0.0)	65 (79.3)	

^a^Chi-square test; ^b^ Fisher’s exact test; *Significance at *p *< 0.05.

In this study, 26 (31.7%) were rheumatologists, 18 (22.0%) were oncologists, 28 (34.1%) were hematologists, and 10 (12.2%) were from other specialties. The majority of respondents were female (53.7%), aged between 25–40 years old (47.6%), with more than or equal to 16 years of experience as physicians (48.8%), self-reported as familiar with biosimilars (53.7%), and had experience prescribing biosimilars (79.3%). The study found that the distribution of gender, self-assessment of familiarity, and previously prescribed biosimilars were different significantly across the specialty types (*p* < 0.05).

### Assumptions assessment for discriminant function analysis

The assumptions assessment for discriminant analysis were performed [23]. Firstly, five factors were tested for univariate normal distribution using Z-value of skewness & kurtosis coefficient. For the medium sample size (50–300), if Z values were within ± 3.29, the data were assumed to be normally distributed [25]. The results showed all variables had normal distribution except *X5* (attitude toward price of biosimilars). However, the Q-Q plots indicated that *X5* to be normal. Moreover, the multivariate normal distribution was also performed using Mahalanobis distances. Its maximum value was 15.776 which was less than 20.52 (critical value; df = 5). It indicated that there was no presence of multivariate outliers. Thus, the normal distribution assumption was acceptable.

Secondly, the equal dispersion matrices were performed using Box’s M test. It showed that there was no significance (*p *= 0.093). Therefore, the matrices of variance were equal. Thirdly, Pearson correlation test showed there was linear relationship between dependent and independent variables. Finally, multicollinearity test was performed. It showed that the tolerance values of all variables were nearly 1, while VIF values were not close to 10. Therefore, there was no multicollinearity issue. In summary, the dataset of all variables seems to meet all assumption criteria, so the discriminant function analysis can be used.

### Descriptive statistics of independent variables related to biosimilar prescribing behaviors

Table 2 shows the descriptive statistics of five independent variables (*X1*-*X5*) among two groups of respondents: Group 1 (non-prescribing biosimilars) and Group 2 (prescribing biosimilars).

**Table 2:** Mean scores between two groups of respondents in the familiarity and attitude domains.

Variables	Group 1: non-prescribing biosimilars (n = 17)		Group 2: prescribing biosimilars (n = 65)		t-value	*p*-value
	Mean score (%)	SD	Mean score (%)	SD		
*X1*	50.27	20.86	62.24	14.92	−2.698	0.008*
*X2*	80.88	18.29	67.89	21.65	2.270	0.026*
*X3*	47.06	31.58	38.15	23.64	1.286	0.202
*X4*	52.94	37.38	45.39	27.56	0.931	0.354
*X5*	52.94	29.01	41.54	21.27	1.518	0.144

**Note.** *X1* = Familiarity, *X2* = Attitude towards biosimilar medications, *X3* = Attitude towards scenario of biosimilars’ prescribing, *X4* = Attitude toward naming biosimilars in prescriptions, *X5* = Attitude towards price of biosimilars; *Significance at *p* < 0.05.

Group 2 had a total of 65 respondents which was higher than Group 1. The mean scores for all variables in Group 2 were lower than in Group 1 except the *X1*. The 2-sample t-test revealed significant differences of mean scores between Group 1 and Group 2 on the variables *X1* and *X2* (*p* < 0.05).

### Stepwise discriminant function equations

Stepwise method was used to select the best independent variables from all five variables in this study. The results of DFA were summarized in Table 3.

**Table 3:** Summary of canonical discriminant functions.

Variables	Unstandardized coefficient	Standardized coefficients	Structure matrix (rank)
***X1***	0.049	0.804	0.716 (1)
***X2***	−0.033	−0.703	−0.603 (2)
**Constant**	−0.590		
**Canonical correlation**	0.388		
**Wilks’ lambda**	Wilks’ lambda = 0.849, Chi-square = 12.896, *p *= 0.002		

**Note.** *X1* = Familiarity, *X2* = Attitude towards biosimilar medications.

The structure matrix indicated that *X1* was the most important variable for the discriminate function followed by *X2*. The other variables did not differentiate between prescribed and non-prescribed biosimilar groups. Moreover, the standardized coefficient confirms that *X1* had the strongest positive relationship with the dependent variable compared to *X2*, whereas *X2* indicated a strong negative relationship. Therefore, the discriminant function for this study can be described by the following equation.

*Y* = (0.049 * *X1*) + (– 0.033 * *X2*) + (-0.590)

where Y = Discriminant function score, *X1* = Familiarity, *X2* = Attitude toward biosimilar medications

The Wilks’ lambda and Chi-square results confirmed that these two factors could explain the differences between the respondents who prescribed and those who did not prescribe biosimilars significantly (*p* = 0.002). By squaring canonical correlation, it indicated that these factors could explain 15.1% of the variation between groups.

**Fig 1:**
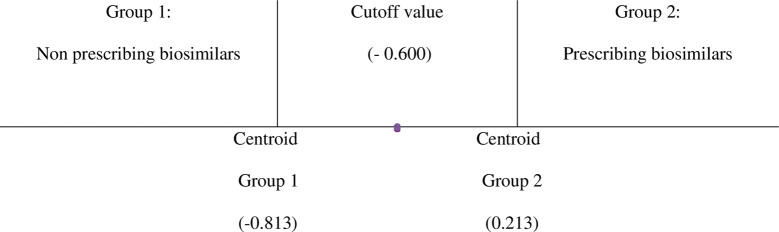
The visual representation of cutoff value.

Fig 1 shows that the cutoff value was −0.600 which was the decision point for group classification. When the Y-value was above −0.600, the individual would be classified in Group 2 (Prescribing biosimilars), otherwise the individual would be classified in Group 1 (Non-prescribing biosimilars).

### Predictive accuracy of equation

Regarding the data of the original group in Table 4, the overall accuracy rate (Hit ratio) was 82.9%.

**Table 4:** Classification results of equation.

Classification	Predicted Group Membership		Total
	Group 1 (%)	Group 2 (%)	
**Original**			
** **Group 1	4 (23.5%)	13 (76.5%)	17 (100%)
** **Group 2	1 (1.5%)	64 (98.5%)	65 (100%)
**Cross-validated**			
** **Group 1	2 (11.8%)	15 (88.2%)	17 (100%)
** **Group 2	2 (3.1%)	63 (96.9%)	65 (100%)

**Note:** 82.9% of original grouped cases correctly classified.

79.3% of cross-validated grouped cases correctly classified.

Group 2 were classified with better accuracy (98.5%) than Group 1 (23.5%). Press’s Q statistic was 35.56 which exceeded the critical value (6.63). It indicated that the predictive accuracy rate of this equation was statistically better than chance. However, for the cross-validation, the equation showed a slightly lower accuracy rate of 79.3%. When calculated Press’s Q, the value was 28.10. Thus, it indicated that the prediction power was acceptable.

### Summary of results

The results from the above-mentioned analysis were summarized as follows:

1. The stepwise discriminant function equation of this study was “*Y* = (0.049 * *X1*) + (– 0.033 * *X2*) + (-0.590)”; where *Y *= Discriminant function score, *X1 *= Familiarity, *X2* = Attitude toward biosimilar medications.
2. Familiarity (*X1*) and attitudes toward biosimilar medications (*X2*) were significantly important for the classification or prediction of group membership, with familiarity factor having a strong positive effect and attitudes toward biosimilar medications having a strong negative effect.
3. Familiarity factor had the most significant influence on group classification, followed by attitude toward biosimilar medications.
4. Based on the equation, 15.1% of the variance between groups can be explained by familiarity (*X1*) and attitude toward biosimilar medication factor (*X2*).
5. Overall, this equation indicates significant discriminatory power.
6. Based on the equation, the decision point for group classification was −0.600.
  6.1. If the discriminant score was < −0.600, the member will be classified as Group 1 (Non-prescribing biosimilars).
  6.2. If the discriminant score was > −0.600, the members will be classified as Group 2 (Prescribing biosimilars)
7. The overall accuracy rate of this equation was 82.9%. It can predict or identify non-prescribing physicians of biosimilars (Group 1) with an accuracy of 23.5%, while it can correctly predict or identify physicians who prescribe biosimilars (Group 2) with an accuracy of 98.5%.

## Discussion

Global uptake of biosimilars continues to rise [1]. Due to the current economic crisis, the rapid growth in the biosimilar market is driven by the several demand-side and supply-side policies for biosimilars to control healthcare expenditure and expand patient access to biologic treatments [4]. Understanding the factors that influence biosimilar prescribing among physicians could inform the development of demand-side policies, which may have a long-term impact on biosimilar uptake.

According to our knowledge, this is the first study using DFA to predict group memberships among Thai physicians (prescribed and non-prescribed biosimilar groups). The study aimed to classify both groups by using psychological factors obtained from the online survey and identify the important factors that impact the decision making during prescribing practice. Before applying for DFA, it is essential to ensure that all assumptions are met to support the validity of the model, as violations of these assumptions can affect its performance [23]. Regarding our study, the dataset of familiarity and attitude towards biosimilars met all necessary assumptions for DFA. Thus, the application of DFA was considered appropriate and more suitable for distinguishing between physician groups in this context than alternative methods such as logistic regression.

This study collected five variables from the survey, namely familiarity, attitude toward biosimilar medications, attitude toward scenario of biosimilars’ prescribing, attitude toward naming biosimilars in prescriptions, and attitude toward price of biosimilars. Although some variables did not show statistically significant differences of mean scores between both groups by using the univariate analysis, all variables were still included in DFA to evaluate the impact in overall. The stepwise DFA was employed to identify the most influential factors related to group classification. In this study, one discriminant function was developed. Wilks’ lambda showed significant value (*p* < 0.05). It indicated the ability of this function can significantly classify the group membership in this study.

Regarding the analysis, it revealed two influential factors, namely familiarity and attitude towards biosimilar medications factors that could discriminate the group memberships. The finding was consistent with the concept of Knowledge/Attitude/Practice (KAP) [26] and the Theory of Planned Behavior [27,28] which described the linkage of psychological factors generating human behavior. For the familiarity factor, it was the most important predictive factor that can classify the biosimilar’s prescribing behaviors. In this study, it implies that the higher familiarity, the higher possibility to classify as the biosimilar prescribers. Our results aligned with the previous survey studies, which found that the necessity of education or physician training to get used to biosimilars would improve the willingness to prescribe biosimilars [12–15]. Furthermore, the attitude towards biosimilars medications was considered as the second strongest to differentiate both groups. This finding was consistent with other studies that found attitude towards biosimilars would relate to the biosimilar prescribing [12,29,30]. However, this factor would likely predict non-prescribing biosimilars. The reason behind this may be attributed to the reluctance of prescribe due to other concerns about biosimilars. Further study related to concerns of using biosimilars should be investigated.

Previous survey studies conducted in Korea and the United States reported other factors, such as cost or pricing of biosimilars and specific prescription scenarios, as influential on the physicians’ willingness to prescribe biosimilars [29,31]. These studies primarily employed descriptive analysis, chi-square tests, or t-tests to assess the association between variables. However, these factors were not strong enough to discriminate between groups in our study. This suggests that differences in statistical methods and study datasets may partly account for the variation in finding. Furthermore, our study found that attitudes toward naming biosimilar were not important. This finding also aligned with other studies that showed these factors have little or no impact on the prescribing of biosimilars [12,30,31].

According to the Theory of reasoned action and planned behavior [28], the background factors such as demographic characteristics, and socioeconomic status can influence behavior indirectly through their effects on behavioral, normative, and control beliefs. These beliefs subsequently affect attitudes, subjective norms, and perceived behavioral control, ultimately contributing to the formation of behavioral intentions. Although this study did not directly examine the association between demographic characteristics and biosimilars prescribing experience, this theory aligns with finding from another secondary analysis study using the same dataset with this study, which showed that medical specialty and self-assessment familiarity were significantly associated with biosimilar prescribing [32]. This suggests that these variables may indirectly influence prescribing behavior through their impact on discriminant factors, including familiarity and attitude toward biosimilar medications, in this study. However, it found no significant relationship between other demographic variables – including gender, age, and years of physician’s experience – and physicians’ experience in prescribing biosimilars.

Furthermore, the uptake of biosimilars is influenced not only by physician-related factors but also by various external factors. Several studies have identified the potential contributors to biosimilar adoption, including hospital or national policies and guidelines, insurance coverage, patient demographics and acceptance, economic incentives, regulatory support and the influence of key opinion leaders [12,33–39]. These external factors may play a pivotal role in shaping physicians’ decisions and promoting biosimilar use.

The cutoff value of this study was −0.600 which could help to segment or predict respondents who would be more likely to prescribe biosimilars from those who would not. However, this cutoff value was derived from Thai physician data and reflects the point that separates higher and lower likelihood of prescribing biosimilars based on familiarity and attitude. In practice, it can help identify which specific factors should be improved to shift a physician’s score above the threshold – for example, enhancing familiarity or trust in biosimilars. This can guide the design of targeted policies or interventions. However, this value is context-specific; other countries would need to determine their own cutoffs based on local data. Although the sample size in this study was small, the predictive accuracy of this model was high at 82.9% overall. The cross validation confirmed that the model was still powerful. Even though the accuracy rate was a bit lower than the original dataset, it was still acceptable at nearly 80%.

Overall, the discriminant function obtained from this study was simple with 2 keys predictive factors, easy to interpret and useful for the policymakers. To increase biosimilars uptake, it could be applied to develop measures such as educational programs or training to increase familiarity with biosimilars to cover all essential topics such as drug characteristics, regulatory aspect, efficacy and safety profiles, extrapolated indication, and interchangeable concept. Whereas improving attitude towards biosimilar medications together with educational support would still be required, although these factors may not have enough potential to influence biosimilars’ prescribing behavior.

### Strengths and limitations

One of the key strength of this study was its use of discriminant function analysis, a powerful tool for group classification when its assumptions are met. Additionally, it is effective even with small sample sizes, making it a valuable method for this research.

However, there are some limitations to consider. Firstly, the study experienced a lower response rate than expected from the online survey, resulting in a smaller sample size. This reduced sample size may have increased the risk of type II error, as the reduced statistical power lowers the model’s ability to detect the potential discriminant factors in the population. Nevertheless, the available data were considered suitable for conducting DFA, and the key findings were both statistically valid and practically meaningful. Moreover, the cross-validation technique was used to assess the robustness of the classification model. However, the results should be interpreted with caution, particularly in terms of their generalizability, as the sample groups were medical specialists in rheumatologists, oncologists and hematologists who mainly prescribe biological drugs. Thus, the findings may not fully represent the all specialist physicians in Thailand.

Secondly, since the study was based on the online voluntary survey using convenience sampling approach, there might have been non-response bias. Physicians who did not use biosimilars might choose to ignore the survey, which meant the findings were not reflecting the views of the populations.

Thirdly, due to unequal group sizes between the non-prescribed and prescribed biosimilars groups, this analysis set the prior probability based on group size [23]. Using group size-based prior probabilities can accurately reflect the classification of sample data. However, the finding might not generalize well to larger populations where group proportions differ from those in this study.

Fourthly, although the assumptions of DFA were met, the model remains data-dependent and may not generalize to other populations or settings. In addition, potential endogeneity bias may result from unmeasured factors that influence both predictors and outcomes. These factors may include external factors such as patient characteristics, and healthcare entitlements. Such bias can affect the validity of the results. Further research should validate the model with new datasets and account for possible confounders to enhance external validity and model robustness. Nevertheless, the methodology used in this study – particularly the application of DFA as a classification method – offers practical value and may be adapted in similar research contexts. Countries with resource limitations similar to Thailand may find this method especially applicable, as it provides a structured way to identify key differentiating factors even in the absence of large-scale data or experimental designs.

Fifthly, this study may be subjected to self-report bias because the respondents were asked to assess their own familiarity with biosimilars. They may have overestimated or underestimated their familiarity due to recall inaccuracies or a desire to appear knowledgeable. Such bias may reduce the discriminative power of the DFA model.

For future research, it would be valuable to expand the sample to include a broader range of specialists and general practitioners, allowing the findings to be more broadly applicable to all physicians in Thailand. Additionally, providing incentives for participants could help boost the response rate. To reduce the risk of selection bias, employing random sampling methods would also be beneficial.

## Conclusion

Discriminant function analysis can be effectively used to categorize the groups of biosimilar practices among physicians. This method could also be used to identify the key factors namely familiarity and attitude toward biosimilar medications which are most fitted with data to discriminate whether the physicians prescribe or not prescribe biosimilars. However, other external factors such as policies, insurance coverage, patient demographics and acceptance, economic incentives, regulatory support and the influence of key opinion leaders are also likely to play a substantial role. The findings from this study offer valuable insights that can inform the development of policies and measures aimed at enhancing biosimilar uptake in both the private and public healthcare sectors. Nevertheless, it is important to consider that this study focused on specific factors; therefore, integrating these findings with additional research on other influencing elements – such as economic considerations, regulatory environments, and patient perceptions – will provide a more comprehensive foundation for policy development. Such a holistic approach will better support the effective and sustainable integration of biosimilars into clinical practice.
